# Elevated Thresholds for Light Touch in Children With Autism Reflect More Conservative Perceptual Decision-Making Rather Than a Sensory Deficit

**DOI:** 10.3389/fnhum.2020.00122

**Published:** 2020-04-07

**Authors:** Jennifer M. Quinde-Zlibut, Christian D. Okitondo, Zachary J. Williams, Amy Weitlauf, Lisa E. Mash, Brynna H. Heflin, Neil D. Woodward, Carissa J. Cascio

**Affiliations:** ^1^Graduate Program in Neuroscience, Vanderbilt University, Nashville, TN, United States; ^2^Graduate Program in Biostatistics, Georgia State University, Atlanta, GA, United States; ^3^Medical Scientist Training Program, Vanderbilt University School of Medicine, Nashville, TN, United States; ^4^Frist Center for Autism and Innovation, Vanderbilt University, Nashville, TN, United States; ^5^Department of Pediatrics, Vanderbilt University Medical Center, Nashville, TN, United States; ^6^San Diego Joint Doctoral Program in Clinical Psychology, San Diego State University/University of California, San Diego, CA, United States; ^7^Graduate Program in Clinical Psychology, Florida International University, Miami, FL, United States; ^8^Department of Psychiatry and Behavioral Sciences, Vanderbilt University Medical Center, Nashville, TN, United States; ^9^Vanderbilt Kennedy Center, Vanderbilt University, Nashville, TN, United States

**Keywords:** autism, tactile, psychophysics, response bias, signal detection

## Abstract

Individuals with autism spectrum disorder (ASD) are often behaviorally hyper-reactive to light touch, but it is unclear to what degree this arises from a fundamental sensory difference vs. higher order systems for attention or emotion processing. Thus far, experimental findings for light touch detection are mixed, and few previous studies have independently considered sensitivity (the ability to discriminate signal from noise) and decision criterion (the overall response bias or tendency to answer “yes” or “no” in a detection task). We tested a large sample of children, adolescents, and adults with ASD (*n* = 88) and with neurotypical (NT) development (*n* = 59) using von Frey filaments to derive light touch thresholds at the palm. We calculated signal detection metrics for sensitivity (A_z_) and response criterion (*c*) from hit and false alarm rates. Both metrics exhibited significant group differences, such that the ASD group was less sensitive, but had a much more conservative response criterion. We used a best subset model selection procedure in three separate ordinal regressions for the whole group, adults, and children/adolescents. In all selected models, *c* was by far the most significant predictor of threshold, supplanting effects of diagnostic group that were significant in the baseline models. In contrast, Az was not a significant predictor of threshold in any of the models. Mean values of *c* were similar for adults with and without autism and for children/adolescents with ASD, but lower (more liberal) in neurotypical children/adolescents. This suggests that children with ASD exhibit a conservatism in their perceptual decision-making that differs from their NT peers but resembles that of adults. Across the sample, the value of *c* was significantly and positively correlated with age and with autism symptoms (SRS-2 total score), in addition to thresholds. The results of this study suggest that, rather than a sensory difference in detection of light touch, there is a difference in response bias such that children with ASD are more conservative/likely to report “no” if unsure, than their young NT peers. Future work should consider the implications of conservative response criterion in ASD for commonly used forced-choice psychophysical paradigms.

## Introduction

Autism Spectrum Disorder (ASD) is characterized by impairments in the social-communication and stereotypical behavior domains (American Psychiatric Association, 2013). Abnormal responses to sensory stimuli are an extremely commonly reported behavioral feature of ASD, prompting the addition of “hypo- or hyper-reactivity to sensory input” as part of the repetitive behavior domain in the DSM-5 (American Psychiatric Association, 2013). Sensory abnormalities in ASD present themselves in varying patterns across multiple modalities like *hypo*-reactivity to sound but *hyper*-reactivity to touch (Baranek et al., 2006). In the somatosensory domain, some of the abnormal sensory responses like hypersensitivities to innocuous, light touch, and textures are thought to be linked to altered sensory thresholds, but the evidence for a link between aberrant behavioral reactivity to touch and sensory detection thresholds is very limited. One problem is that there is a lack of behavioral measures that assess hypo- or hyper-reactivity in a single sensory modality. Thus, these measures reflect the multisensory heterogeneity described above, in which different sensory modalities may contribute to the same behavioral reactivity pattern. Additionally, traditional sensory questionnaire measures rely on assumptions that differences in reactivity to sensory stimuli are caused by differences in sensitivity. The complexity of behavioral reactivity, encompassing sensory, perceptual, emotional, and cognitive factors, may not map cleanly onto pure sensory detection ability as measured by psychophysical paradigms.

Several studies have, however, tested the hypothesis that tactile detection thresholds are altered in ASD. Vibrotactile stimuli are commonly used for tactile detection because of their precision, potential for automation, and direct mapping to specific classes of peripheral mechanoreceptors. However, these stimuli do not map directly to the daily experiences of touch that are most commonly reported to be problematic for individuals with ASD (Baranek and Berkson, 1994). Because of the common complaint that light touch specifically is perceived as aversive, it may be important to examine this separately from other types of stimuli. Cascio et al. (2008) reported no differences in light touch contact detection threshold at the dorsal forearm or palm using von Frey filaments in a small sample of adults with autism, a negative result replicated by Fründt et al. (2017) and Fukuyama et al. (2017) in larger adult samples. However, elevated thresholds for light touch *were* reported in the largest sample of adults with autism to date (Vaughan et al., 2019). In children, an early study (O’Riordan and Passetti, 2006) reported intact contact detection on the volar forearm in a small sample; 10 years later Riquelme et al. (2016) reported *lower* thresholds at the face and dorsum of the hand in children with ASD. While this finding could be consistent with a sensory basis for hyper-reactivity to light touch, behavioral reactivity was not reported in this study.

Further, the Riquelme et al. (2016) study reported a lack of group differences on the palmar surface of the hand. While not a primary somatic site for hyper-reactivity, the palmar surface is densely innervated with receptors for light touch and is highly relevant for fine motor ability (McGlone et al., 2014), another domain that is often affected in individuals with ASD (Jeste, 2011; Chukoskie et al., 2013). For example, sensory ability on the palm contributes to grip and manual dexterity, both of which have been reported to be altered in individuals with ASD (MacDonald et al., 2014; Wang et al., 2015; Travers et al., 2017). Notably, fine motor skills in high risk infants predict later outcomes in expressive language (Choi et al., 2018) and autism symptom severity (Iverson et al., 2019). These findings highlight the potential for deficits in very low level sensory and motor abilities to “cascade” into deficits in the higher-level behaviors implicated in ASD.

Methods for ascertaining sensory detection thresholds vary widely and also likely play a role in discrepant findings. Highly efficient adaptive staircase methods, for example, facilitate assessment of sensation across a wider range of attentional capability than classic methods such as the method of constant stimuli (Treutwein, 1995). Thus, sample characteristics and data quality may covary with the methodological decisions made across studies. Few previous psychophysical studies in ASD have utilized signal detection theory (SDT), which allows the separation of sensitivity [the ability to discriminate signal (e.g., touch) from noise (e.g., no touch), and response criterion (degree of overall individual bias toward or away from responding “yes” when unsure] (Green and Swets, 1966; Stanislaw and Todorov, 1999). When using yes/no decision tasks to assess sensory detection, SDT approaches afford clarity on sensory vs. decisional processes that may be related to higher order cognitive phenomena. Studies of perceptual decision making in the visual system indicate that participants with ASD are slower and more cautious in their responses in a two-alternative forced choice task (Pirrone et al., 2018), suggesting they prioritize accuracy over speed.

Thus, characterizing the mechanisms of somatosensory dysfunction in ASD has been limited by mixed findings that are likely the result of differing stimuli, methods, and sample characteristics, including the size, age range, and symptom profiles of participants. The paucity of light touch literature using a single standardized method across a wide range of ages and large sample size highlights a gap in the empirical research that warrants further investigation of light touch in ASD. Further, our understanding would benefit from more psychophysical studies designed to separate sensory from decisional differences. To this end, the current study employs standardized psychophysical methods and signal detection theory to characterize light touch detection in a large sample of children and adults diagnosed with ASD.

## Materials and Methods

### Participants

#### Children/Adolescents

Participants included 90 children and adolescents between the ages of 7 and 17 years: 35 neurotypical children/adolescents (*M_age_* = 9.49 years, *SD* = 2.61 years; 9 female) and 55 children/adolescents with autism (*M_age_* = 10.91 years, *SD* = 3.35 years; 9 female). Groups did not differ in gender but exhibited significant differences in age and FSIQ. Participant characteristics are provided in Table 1 (whole sample) and Table 2 (child/adolescent subsample).

**TABLE 1:** Descriptive statistics and group comparisons for whole sample.

**Variable**	**N (ASD/NT)**	**ASD (Mean, *SD*)**	**NT (Mean, *SD*)**	**δ (95% CI)**	***P*-value**
Age (years)	88/59	17.40 (10.35)	17.20 (10.07)	−0.035(−0.229,0.162)	0.729
Verbal IQ	84/58	101.42 (16.40)	109.81 (14.17)	0.313(0.126,0.478)	**<0.001**
Performance IQ	84/58	105.27 (17.24)	110.79 (17.36)	0.169(−0.028,0.353)	0.088
Full-scale IQ	83/58	104.51 (15.92)	111.69 (15.83)	0.257(0.064,0.432)	**0.007**
SRS-2 total T-score	78/47	73.09 (10.30)	44.26 (6.10)	−0.985(−0.995,−0.957)	**<0.001**
Average thresholds (g)	88/59	0.103 (0.09)	0.065 (0.06)	−0.263(−0.433,−0.075)	**0.005**
Az	86/57	0.874 (0.07)	0.905 (0.08)	0.246(0.046,0.427)	**0.013**
*c*	86/57	0.765 (0.28)	0.613 (0.23)	−0.339(−0.504,−0.15)	**<0.001**

*IQ, Intelligence Quotient; SRS-2, Social Responsiveness Scale-2; Az, sensitivity; c, response criterion. P-values in bold indicate statistically significant differences between groups.*

**TABLE 2:** Descriptive statistics and group comparisons for child/adolescent subsample.

**Variable**	**N (ASD/NT)**	**ASD (Mean, *SD*)**	**NT (Mean, *SD*)**	**δ (95% CI)**	***P*-value**
Age (years)	55/35	10.91 (3.35)	9.49 (2.61)	−0.242(−0.457,−0.001)	**0.043**
Verbal IQ	54/35	100.50 (17.87)	111.34 (12.80)	0.421(0.189,0.608)	**<0.001**
Performance IQ	54/35	105.06 (15.27)	112.94 (18.16)	0.264(0.007,0.489)	**0.037**
Full-scale IQ	54/35	103.50 (16.26)	114.29 (15.48)	0.397(0.155,0.597)	**<0.001**
SRS-2 total T-score	53/29	73.87 (9.92)	43.72 (5.97)	−0.988(−0.997,−0.955)	**<0.001**
SP low registration	42/28	54.02 (10.02)	69.32 (4.23)	0.887(0.747,0.952)	**<0.001**
SP seeking	42/28	94.48 (14.96)	110.32 (12.61)	0.579(0.319,0.758)	**<0.001**
SP sensitivity	42/28	69.67 (13.86)	88.18 (7.32)	0.739(0.539,0.86)	**<0.001**
SP avoiding	42/28	98.69 (14.70)	122.36 (10.22)	0.832(0.668,0.92)	**<0.001**
Average thresholds (g)	55/35	0.095 (0.09)	0.043 (0.03)	−0.417(−0.606,−0.182)	**<0.001**
Az	53/35	0.88 (0.07)	0.884 (0.09)	0.04(−0.217,0.293)	0.761
*c*	53/35	0.761 (0.274)	0.553 (0.191)	−0.44(−0.627,−0.205)	**<0.001**

*IQ, Intelligence Quotient, SRS-2, Social Responsiveness Scale-2; SP, Sensory Profile (caregiver version); Az, sensitivity; c, response criterion. P-values in bold indicate statistically significant differences between groups.*

#### Adults

Participants included 57 adults between the ages of 18 and 54 years: 24 neurotypical adults (*M_age_* = 28.46 years, *SD* = 4.77 years; 10 female) and 33 adults with ASD (*M_age_* = 28.21 years, *SD* = 8.92 years; 12 female). Groups did not differ significantly in age, gender, or full-scale IQ (FSIQ) of the Wechsler Abbreviated Scale of Intelligence, 2nd edition (WASI-II). Participant characteristics are provided in Table 1 (whole sample) and Table 3 (adult subsample).

**TABLE 3:** Descriptive statistics and group comparisons for adult subsample.

**Variable**	**N (ASD/NT)**	**ASD (Mean, *SD*)**	**NT (Mean, *SD*)**	**δ (95% CI)**	***P*-value**
Age (years)	33/24	28.21 (8.92)	28.46 (4.77)	−0.15(−0.157,0.431)	0.331
Verbal IQ	30/23	103.07 (13.48)	107.48 (16.05)	0.12(−0.197,0.414)	0.455
Performance IQ	30/23	105.67 (20.60)	107.52 (15.89)	−0.009(−0.317,0.301)	0.957
Full-scale IQ	29/23	106.38 (15.37)	107.74 (15.86)	−0.024(−0.336,0.292)	0.885
SRS-2 total T-score	25/18	71.44 (11.09)	45.11 (6.39)	−0.985(−0.995,−0.916)	**<0.001**
AASP low registration	25/20	42.76 (11.50)	24.80 (3.78)	−0.846(−0.949,−0.583)	**<0.001**
AASP seeking	26/20	38.50 (5.80)	44.25 (6.05)	0.517(0.171,0.75)	**<0.001**
AASP sensitivity	26/20	47.08 (11.57)	33.90 (3.93)	−0.696(−0.867,−0.378)	**<0.001**
AASP avoiding	26/20	49.46 (14.05)	33.40 (7.36)	−0.69(−0.863,−0.375)	**<0.001**
Average thresholds (g)	33/24	0.116 (0.09)	0.098 (0.07)	−0.072(−0.363,0.231)	0.645
Az	33/22	0.865 (0.07)	0.938 (0.04)	0.594(0.311,0.78)	**<0.001**
*c*	33/22	0.771 (0.29)	0.708 (0.25)	−0.163(−0.451,0.156)	0.311

*IQ, Intelligence Quotient, SRS-2, Social Responsiveness Scale-2; AASP, Adolescent and Adult Sensory Profile (self-report version); Az, sensitivity; c, response criterion. P-values in bold indicate statistically significant differences between groups.*

#### Recruitment and Characterization

Participants were recruited from the community through flyers and from university and medical center autism databases. Autism diagnoses were confirmed by administration of the Autism Diagnostic Observation Schedule-2 (ADOS-2; Lord et al., 2012) and a parent interview including the algorithm items from the Autism Diagnostic Interview-Revised (ADI-R; Lord et al., 1994), when a parent was available to report. The tests were administered and scored by a research-reliable assessor under the supervision of a licensed clinical psychologist specializing in autism diagnostic assessment. Participants in the autism cohort were screened for comorbid psychiatric diagnoses, neurological disorders, and sensory impairments that affect somatosensation. Behavior and co-occurring psychiatric conditions were screened for using parent and guardian reports for children and adolescents, and by self-report for adults. For a subset, the Child Behavior Checklist (CBCL) for children, and the associated Adult Self Report form from the Achenbach System of Empirically Based Assessment (Achenbach, 2015) were used as additional screening tools. The NT group was additionally screened for autism-related traits using the Social Communication Questionnaire (SCQ; Rutter et al., 2003).

Exclusion criteria for both groups included full scale IQ (FSIQ) scores lower than 70 as assessed by the Wechsler Abbreviated Scale of Intelligence-II (WASI-II; Weschsler, 1999), and presence of other neurological and genetic disorders, or sensory impairments not related to ASD etiology. Individuals in the NT cohort were excluded if they had a previous psychiatric history, cognitive or sensory impairment, use of psychotropic medications, or clinically elevated scores on the SCQ. When appropriate, parents of participants in both the NT and ASD groups completed the sensory profile (SP; Dunn, 1999) questionnaire, a 125-item caregiver report on the frequency of sensory experience-related behaviors. Notably, items are scored on a 5-point Likert scale with *lower* scores indicating higher frequency of abnormal behavior. A shorter, self-report version of the sensory profile, the Adolescent/Adult Sensory Profile (AASP; Brown et al., 2001), was administered to the adult groups. The AASP is a 60-item questionnaire assessing attitudes and behaviors related to sensory processing in individuals 11 years and older. Unlike the caregiver SP, higher scores on the 5-point Likert scale indicate higher frequency of abnormal behavior. Written informed consent or assent forms were signed by all participants, and consent was obtained from parents or guardians for minors. Upon study completion, participants were compensated $20 per hour of their time. All procedures were approved by the institutional review board for human subjects at the university medical center.

### Measures

Adult participants in both NT (*n* = 26, mean age = 28.46) and ASD (*n* = 20, mean age = 28.21) groups completed self-report questionnaires measuring autistic traits (SRS-2: Adult Self-Report; Constantino and Gruber, 2012) and sensory features (Adolescent/Adult Sensory Profile; Brown et al., 2001). Parents or guardians of children/adolescents in both groups completed the analogous caregiver-report questionnaires (NT: *n* = 35, mean age = 9.49; ASD: *n* = 55, mean age = 10.91), the SRS-2 school age form (Constantino and Gruber, 2012) and Sensory Profile (Dunn, 1999). SRS-2 total scores were converted to T-scores (*M* = 50, *SD* = 10) to facilitate comparison across the different groups. Scores on the SP and AASP are reported in four “Sensory Quadrants” representing different patterns of responsiveness to sensory stimuli: low registration, sensory seeking, sensory avoiding, and sensory sensitivity. For both the AASP/SP and the SRS-2, self-report for adults and caregiver-report for children were chosen based on the availability of validated measures for that age range.

### Tactile Thresholds

The tactile threshold task was administered as part of a larger battery of experiments, some of which are reported elsewhere (Williams et al., 2019). Tactile stimulation was administered using 10 Von Frey filaments ranging in diameter and associated applied forces from 0.008 to 2.0 g. A method of limits approach was employed to determine thresholds for perceiving touch. The stimuli were administered in four alternating blocks of ascending and descending trials. Whether the session began with an ascending or a descending block was counterbalanced across participants. The participant’s right arm from elbow to fingertips was screened from view using a cardboard panel and a cloth drape. Each trial began with the verbal prompt “Pay attention now” and the filament was lowered to make contact with the thenar eminence of the right palm, until the filament buckled. The participants were then asked to respond either positively or negatively to having felt the stimulus.

Descending blocks began with the 2.0 g filament with subsequent filaments decreasing in diameter until the participant responded negatively to two consecutive trials. Filament mass values before and after the first of two consecutive negative responses were recorded to calculate tactile thresholds for descending blocks. Ascending blocks began with the 0.008 g filament with subsequent filaments increasing in diameter until the participant responded positively to two consecutive trials. Catch trials (trials with no stimuli), were included to allow for the calculation of false alarm rate. Participants were notified of false positive responses before proceeding to the next trial. Three participants were excluded from analyses for meeting the criterion of giving positive responses on more than three catch trials in any block of 10 trials. To account for higher response bias toward “yes” in descending relative to ascending blocks, catch trials were distributed unevenly, with three catch trials in each ascending and seven in each descending block.

Participant tactile thresholds were calculated after completion of two ascending and two descending blocks. The trial values before and after the first two consecutive negative or positive responses that ended a block were averaged across the four blocks to generate an overall tactile detection threshold per participant (as in Cascio et al., 2008). Pooling of trials across blocks was supported by moderate intraclass correlations between the estimated threshold values across each of the four blocks, *ICC* (3.4) = 0.69, 95% CI [0.59, 0.76].

### Signal Detection Metrics

#### Sensitivity

We used the parametric measure *A*_z_ to estimate sensitivity, which has optimal statistical properties and is a monotonic transformation of the more commonly used *d*’ index (Verde et al., 2006). *A*_z_ equals the area under a receiver operating characteristic (ROC) curve that represents the plot of hits vs. false alarms at a constant sensitivity level and different degrees of response bias. As with *d’*, *A*_z_ utilizes the parametric assumptions that latent signal and noise distributions are normally distributed with equal variance. Despite the questionable validity of the equal-variance assumption, *A*_z_ has been found to outperform the “non-parametric” SDT sensitivity *A*’ in simulation studies (Verde et al., 2006). *A*_z_ can be calculated from the following equation (Verde et al., 2006):

$$A_{z}=\phi \,\left(\frac{\phi^{-1}\,\left(H\right)-\phi^{-1}\,\left(F\right)}{\sqrt{2}}\right)$$

Where *H* and *F* are the hit and false alarm proportions, and Φ is the standard normal cumulative distribution function. In cases where *H* was 1, we calculated *A*_z_ by replacing this value with the number of hits plus 0.5 divided by the number of stimulus trials plus one. Similarly, when *F* was 0, we calculated *A*_z_ by replacing this value with 0.5 divided by the number of catch trials plus one.

#### Response Criterion

To estimate each individual’s response criterion, we calculated the parametric measure *c*, which represents the standard deviation of the criterion location relative to the zero-bias point in the signal and noise distributions, with positive values reflecting more conservative strategy (minimizing false alarms by favoring a “no” response) and negative values a more liberal strategy (minimizing misses by favoring a “yes” response). *c* is calculated from the following equation (Stanislaw and Todorov, 1999):

$$C=-\frac{\phi^{-1}\,\left(H\right)+\phi^{-1}\,\left(F\right)}{2}$$

### Analysis Plan

#### Group Comparisons

Before controlling for covariates, we conducted group comparisons between the ASD and NT groups. Demographics, IQ (intellectual quotient), tactile threshold, signal detection metrics of sensitivity, and response criterion (*A*_z_ and *c*, respectively) and questionnaire measures of sensory features were compared between the two groups. Further ASD-NT comparisons were done separately for children/adolescent and adult age-defined subgroups. The Pearson chi-square test without continuity correction was used to compare categorical variables between groups. As most continuous variables of interest were substantially skewed and heteroskedastic, we used Cliff’s delta (Cliff, 1993; Feng and Cliff, 2004), a robust, non-parametric effect-size statistic, to examine diagnostic group differences in these measures. Delta estimates the probability that a randomly selected observation from one group is larger than a randomly selected observation from another group, minus the reverse probability. Values of δ range from −1 to 1, with a value of 0 indicating complete overlap of groups and values of −1 or 1 indicating all values in one group being larger than all values in the other. Cliff’s delta values were computed using the *orddom* package (Rogmann, 2013) in the R statistical language (R Core Team, 2017). Spearman rank correlations were used to examine the association between variables in the whole sample, children/adolescents, and adult subsamples. Correlation coefficients were compared between groups using the confidence interval method of Zou (2007), as implemented in the *cocor* R package (Diedenhofen and Musch, 2015). As these zero-order correlations were exploratory and considered preparatory to the regression models, they were not corrected for multiple comparisons.

#### Regression Models

In order to determine the effects of the various behavioral and demographic variables on tactile thresholds, we conducted a multiple regression analysis. Due to the extreme skewness of the tactile threshold variables, initial multiple regression analysis revealed that assumptions for this model were strongly violated. To resolve this issue, we conducted a proportional-odds logistic regression using the cumulative probability model (CPM; Harrell, 2015; Liu et al., 2017), which is appropriate for use with continuous outcomes. The CPM is a semi-parametric regression model that functions as a multi-predictor generalization of the Wilcoxon–Mann–Whitney test. The significance of the model as a whole was tested using a likelihood ratio χ^2^ test comparing the fully specified model to a baseline intercept-only model. Fit of the model to the data was calculated using the Nagelkerke (1991) *R*^2^ index.

Ordinal logistic models were fit in two steps. A baseline model was first fit, in which the tactile threshold was regressed on diagnosis group (ASD vs. NT), age (in years), sex (Male vs. Female), counterbalance order (ascending blocks first vs. descending). In the second step, other predictors (verbal IQ, performance IQ, FSIQ, SRS T-score, SRS sensory item score, *A*_z_, *c*) were added using best-subset regression with the Bayesian Information Criterion (BIC; Schwarz, 1978; Gagné and Dayton, 2002). BIC weights (Wagenmakers and Farrell, 2004) were then used to quantify the probability that the chosen model was the best among all considered models. The approximate Bayes Factor in favor of the best-fitting model over the baseline model (BF_10_) was also calculated from the difference in BIC values between the baseline and best-fitting model. As some of the predictor variables (such as SP and AASP quadrant scores) were only applicable to certain age groups, regression models were also fit separately for the child/adolescent and adult subsamples. SP/AASP quadrants were then also included as potential predictors in the best subset regression models. Due to large correlations between the SP/AASP sensory sensitivity and sensory avoiding quadrants, only the Low Registration, Sensory Seeking, and Sensory Sensitivity quadrant scores were utilized as potential predictors in regression models.

## Results

As a global index of potential tactile detection impairment in ASD, we first compared the tactile thresholds on the entire sample, then, to account for developmental differences in perceptual ability or other variables that may affect thresholds, we conducted separate analyses in the child/adolescent and adult groups. There was a significant group difference in the combined sample, δ = −0.263, *p* = 0.005, with ASD group exhibiting higher threshold.

### Whole Sample Comparisons

The two diagnostic groups were equivalent in terms of age (δ = −0.035, *p* = 0.729), gender [χ^2^(1) = 0.855, *p* = 0.355], and performance IQ (PIQ) (δ = 0.169, *p* = 0.088). Full-Scale IQ (FSIQ) and verbal IQ were significantly lower in the ASD group (FSIQ: δ = 0.257, *p* = 0.007) and (VIQ: δ = 0.313, *p* = 0.001). As expected, large and significant group differences were seen in all questionnaire measures of ASD traits and sensory features. These group differences (*p* < 0.001) were seen in SRS t-score, and all SP self and caregiver quadrant scores (low registration, sensory seeking, sensory sensitivity, and sensory avoiding). Scores on the SP caregiver questionnaire indicated that the ASD group exhibited lower (i.e., more atypical) scores on all four quadrants. AASP scores also indicated that the ASD group had elevated sensory abnormalities in low registration, sensory sensitivity, and sensory avoiding, but scores on the sensation seeking quadrant were more atypical in the NT group.

**FIGURE 1:**
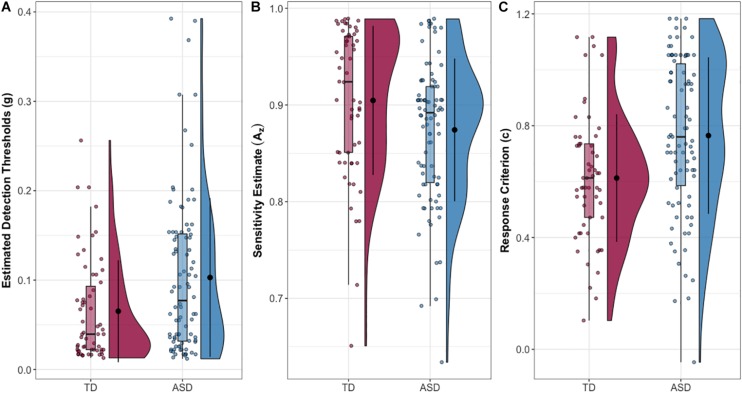
**(A)** Estimated tactile detection thresholds are elevated in individuals with ASD. **(B)** Estimated sensitivity based on hit and false alarm rates (Az). **(C)** Estimated response criterion based on hit and false alarm rates (*c*). Data presented are for the entire sample.

Finally, the ASD and NT group differed in SDT measures of sensitivity (*A*_z_) (δ = 0.246, *p* = 0.013) and response criterion (*c*) (δ = −0.339, *p* = 0.001), with the ASD group exhibiting lower sensitivity (reduced ability to discriminate signal from noise) and higher response criterion (more conservative perceptual decision-making). Descriptive statistics and group comparisons for the whole sample are reported in Table 1 and Figure 1. For a detailed whole sample SDT matrix, see Supplementary Table S1.

### Age-Defined Subgroup Comparisons

**FIGURE 2:**
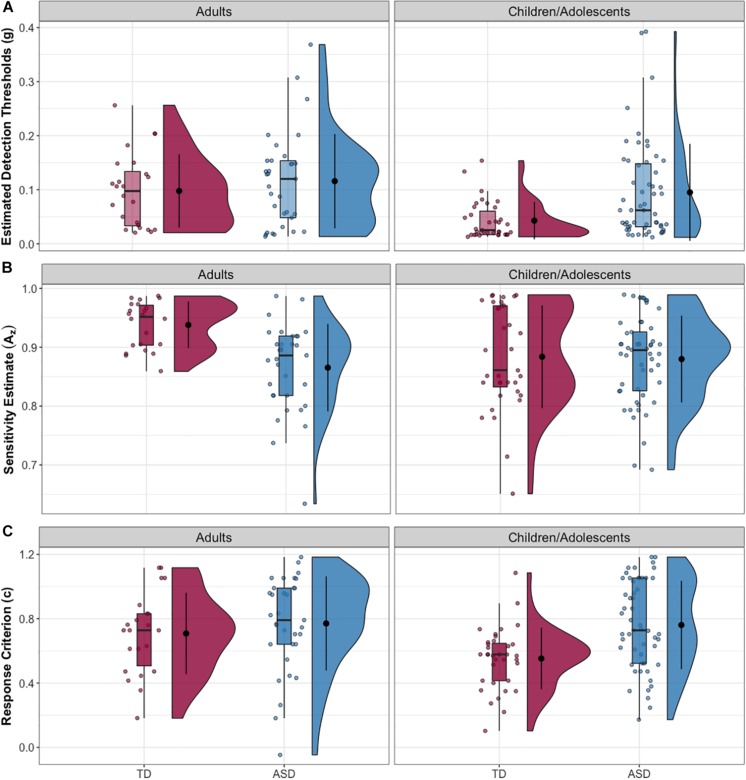
**(A)** Estimated tactile detection thresholds are elevated in individuals with ASD. **(B)** Estimated sensitivity based on hit and false alarm rates (Az). **(C)** Estimated response criterion based on hit and false alarm rates (*c*). Data presented are for the adult subgroup (left) and the child/adolescent subgroup (right).

Full-scale IQ comparisons within age bands revealed that the IQ difference in the whole sample was specific to the child/adolescent group (δ = 0.397, *p* < 0.001), while no FSIQ group differences were found in adults (δ = −0.024, *p* = 0.885). The same trends were found for verbal IQ: child/adolescent group: (δ = 0.421, *p* < 0.001), adult group: (δ = 0.12, *p* = 0.455). The NT child/adolescent group had a significantly higher PIQ than the ASD group, (δ = 0.264, *p* = 0.037), while there was no significant difference in the adult group, (δ = −0.009, *p* = 0.957). The child/adolescent ASD group had significantly higher thresholds than the child/adolescent NT group, (δ = −0.417, *p* < 0.001), while for adults, threshold did not differ between diagnostic groups, (δ = −0.072, *p* = 0.645). The *A*_z_ (sensitivity) difference in the whole sample was limited to the adults (δ = 0.594, *p* < 0.001), while the children/adolescents with and without autism had similar mean values for Az (δ = 0.04, *p* = 0.761). Conversely, the *c* (criterion) difference in the whole sample was driven by the child/adolescents (δ = −0.440, *p* < 0.0001), while the adults with and without autism had similar mean values for *c* (δ = 0.040, *p* = 0.761). Group comparisons for the child/adolescent subgroup are reported in Table 2; group comparisons for the adult subgroup are in Table 3. For both subgroups, group comparisons are depicted in Figure 2. For detailed age group SDT matrices, see Supplementary Table S1.

### Correlational Analyses Between Threshold, Response Criterion, IQ, and Autism Symptoms

Response criterion (*c*) was significantly correlated with threshold (*r*_s_ = 0.607, *p* < 0.001), age *r*_s_ = 0.253, *p* = 0.002, and autism symptom severity (SRS total t score) *r*_s_ = 0.330, *p* < 0.001. In contrast, *A*_z_ was related to thresholds (*r*_s_ = −0.221, *p* = 0.008), but not to age or SRS scores. Neither SDT metric was significantly related to any of the IQ measures. A weak but significant positive correlation was observed between the average thresholds and age (*r*_s_ = 0.309, *p* < 0.001). Another weak correlation was observed between the thresholds and SRS total across the whole sample, *r*_s_ = 0.225, *p* = 0.012. In the child/adolescent subgroup, average thresholds were significantly correlated with SRS total scores (*r*_s_ = 0.389, *p* < 0.001). Adult thresholds did not correlate with SRS total scores (*r*_s_ = −0.006, *p* = 0.970), and a similar pattern was observed between age and SDT metrics (*c*: *r*_s_ = 0.164, *p* = 0.23; Az: *r*_s_ = 0.006, *p* = 0.962). Using Zou’s (2007) confidence interval method, the group difference in threshold-SRS correlations was found to be statistically significant, Δ*r* = −0.395, CI_95_ [−0.740, −0.033]. No association was found between tactile thresholds and any of the IQ measures.

### Correlational Analyses Between Threshold and Sensory Reactivity Measures

AASP-self report quadrants did not correlate with average thresholds, while some of the SP-caregiver quadrants showed some weak association with thresholds. For instance, SP low registration, (*r*_s_ = −0.286, *p* = 0.016), SP sensitivity, (*r*_s_ = −0.325, *p* = 0.006), SP avoiding, (*r*_s_ = −0.300, *p* = 0.011), show weak but significant correlation with average thresholds.

### Regression Models

#### Whole Sample

When fit to the whole sample, the baseline model (estimated threshold regressed on diagnostic group, age, gender, and counterbalance order) fit significantly better than the intercept-only model (Table 4). Of the four baseline predictors, only ASD diagnosis (adjusted odds ratio (*aOR* = 2.30, CI_95_ [1.29, 4.09], *p* = 0.005) and older age (*aOR* = 1.07, CI_95_ = [1.04, 1.10], *p* < 0.001) significantly predicted higher tactile detection thresholds. Per the BIC, the best subset regression analysis revealed a five regressor (baseline plus *c*) model to be the best model (BIC weight = 0.367, BF_10_ = 1.23 × 10^10^). All other candidate predictors (verbal IQ, performance IQ, full-scale IQ, total SRS score, SRS sensory item, *A*_z_, and interaction terms for diagnosis by age and diagnosis by verbal IQ) were not included in the final model. In the final model, older age (*aOR* = 1.06, CI_95_ = [1.03, 1.1], *p* < 0.0001), male gender (*aOR* = 2.06, CI_95_ = [1.06, 4.0], *p* < 0.032), and higher (more conservative) criterion (*c*) (*aOR* = 76.38, CI_95_ = [20.78, 280.7], *p* < 0.0001) significantly predicted higher thresholds (Table 5).

**TABLE 4:** Baseline regression models for tactile detection thresholds.

**Whole sample: baseline model**				**Adults: baseline model**			
**Predictor**	***aOR* (95% CI)**	**Wald χ^2^**	***P***	**Predictor**	***aOR* (95% CI)**	**Wald χ^2^**	***P***
**Diagnosis (ASD)**	**2.30 (1.29, 4.09)**	**8.05**	0.005*	Diagnosis (ASD)	1.12 (0.45 2.81)	0.06	0.807
Sex (Male)	1.66 (0.87, 3.15)	2.38	0.123	Sex (Male)	1.19 (0.46, 3.07)	0.13	0.718
**Age (years)**	**1.07 (0.87, 1.10)**	**17.93**	<0.001*	**Age (years)**	**1.10 (1.01, 1.18)**	**5.32**	0.021*
Counterbalance	0.66 (0.36, 1.18)	1.98	0.159	**Counterbalance**	**0.33 (0.12, 0.88)**	**4.91**	**0.027**
**Model fit**	χ^2^(4) = 28.06	*p* < 0.001*	*R*^2^ = 0.174	**Model fit**	χ^2^(4) = 11.62	*p* = 0.022*	*R*^2^ = 0.184

**Children: baseline model**							
**Predictor**	***aOR* (95% CI)**	**Wald χ^2^**	***P***				

**Diagnosis (ASD)**	**3.83 (1.71, 8.60)**	**10.60**	0.001*				
**Sex (male)**	**2.49 (1.01, 6.17)**	**3.89**	0.049*				
Age (years)	1.00 (0.90, 1.13)	<0.01	0.945				
Counterbalance	0.82 (0.38, 1.77)	0.75	0.621				
**Model fit**	χ^2^(4) = 15.66	*p* = 0.004*	*R*^2^ = 0.160				

*Significant predictors in each model are bolded; aOR, Adjusted Odds Ratio; PIQ, Performance IQ. ^∗^p < 0.05.*

**TABLE 5:** Best-fitting regression models for tactile detection thresholds.

**Whole sample: best-fitting model (Baseline + *c*)**				**Adults: best-fitting model (Baseline + *c*)**			
**Predictor**	***aOR* (95% CI)**	**Wald χ^2^**	***P***	**Predictor**	***aOR* (95% CI)**	**Wald χ^2^**	***P***
Diagnosis (ASD)	1.48 (0.82, 2.66)	1.67	0.196	Diagnosis (ASD)	0.96 (0.38, 2.44)	0.01	0.933
**Sex (male)**	**2.06 (1.06, 4.0)**	**4.6**	**0.032**	Sex (Male)	2.15 (0.8, 5.8)	2.31	0.129
**Age (years)**	**1.06 (1.03, 1.1)**	**14.78**	<0.001*	**Age (years)**	**1.09 (1.01, 1.18)**	**5.27**	0.022*
Counterbalance	0.71 (0.4, 1.28)	1.31	0.253	Counterbalance	0.43 (0.15, 1.18)	2.69	0.101
***C***	**76.38 (20.78, 280.7)**	**42.63**	<0.001*	***c***	**39.28 (4.89, 315.6)**	**11.92**	**<0.001**
**Model fit**	χ^2^(5) = 77.12	*p* < 0.001*	*R*^2^ = 0.408	**Model fit**	χ^2^(5) = 25.10	*p* < 0.001*	*R*^2^ = 0.356

**Children: best-fitting model (Baseline + *c*)**							
**Predictor**	***aOR* (95% CI)**	**Wald χ^2^**	***P***				

Diagnosis (ASD)	2.12 (0.93, 4.85)	3.18	0.075				
Sex (Male)	2.56 (0.93, 7.03)	3.34	0.067				
Age (years)	0.94 (0.83, 1.06)	1.1	0.295				
Counterbalance	0.86 (0.39, 1.88)	0.15	0.701				
***c***	**142.8 (24.11, 845.6)**	**29.88**	**<0.001**				
**Model fit**	χ^2^(4) = 49.52	*p* < 0.001*	*R*^2^ = 0.423				

*Significant predictors in each model are bolded; aOR, Adjusted Odds Ratio. Best fitting model was determined using a best subset regression procedure and Bayesian Information Criterion (BIC). c, response criterion. ^∗^p < 0.05.*

#### Child/Adolescent Subgroup

The baseline regression model (diagnostic group, age, gender, and counterbalance order) in the child/adolescent group again fit significantly better than the intercept-only model (Table 4). In this model, ASD diagnosis (*aOR* = 3.83, CI_95_ [1.71, 8.62], *p* = 0.001) and male gender (*aOR* = 2.49, CI_95_ [1.01, 6.17], p = 0.049) were both significantly associated with elevated tactile detection thresholds. Best-subset regression procedures again revealed that the baseline model plus *c* was the best model per BIC (BIC weight = 0.173, = BF_01_ = 8.68 × 10^6^), and no other predictors were added to the final model. In the final model, only higher criterion (*aOR* = 142.8, CI_95_ [24.11, 845.6], *p* < 0.0001) significantly predicted thresholds (Table 5).

#### Adult Subgroup

The baseline model (diagnostic group, age, gender, and counterbalance order) for the adult group also showed improved fit over an intercept-only model (Table 4). In this model, older age and counterbalance condition, but not ASD diagnosis, significantly predicted tactile detection thresholds. Based on BIC criteria, as in the previous models, the best-subset regression model included C in addition to the baseline predictors (BIC weight = 0.085, BF_01_ = 161.9). In the final model, both older age (*aOR* = 1.09, CI_95_ [1.01, 1.18], *p* = 0.022) and higher criterion (*aOR* = 39.28, CI_95_ [4.89, 315.6], *p* = 0.001) were significant predictors of tactile threshold (Table 5).

## Discussion

In a large sample of individuals with autism that included both children and adults, we found elevated thresholds to light touch at the thenar eminence of the palm. Elevated thresholds in the ASD group were present in the children and adolescents sample and were absent in autistic adults. The autism group also exhibited lower ability to distinguish signal from noise, defined by *A*_z_, and higher response criterion, defined by *c* and reflecting the overall bias toward being conservative and responding “no” when unsure if a stimulus is present. The former difference (sensitivity) was specific to the adult subsample, while the latter (response criterion) was specific to the child/adolescent subsample. The group differences were reflected in the regression models: (1) for the whole sample, older age, male gender, and a higher response criterion significantly predicted elevated thresholds, (2) for the child/adolescent subgroup, only response criterion significantly predicted thresholds while (3) for the adult subgroup, older age and response criterion were significant predictors.

The most robust finding in the current study is the strong influence of response criterion on detection thresholds and the striking differences in how this variable manifested itself across the groups. Criterion was much higher in children and adolescents with ASD relative both to NT peers and to adults. In the baseline model for the children and adolescents, the adjusted odds ratio of the autism diagnosis factor was 3.83, suggesting that elevated thresholds are nearly four times as likely for children and adolescents with autism compared to those without. However, when criterion was added to the model as prescribed by the BIC, there was no longer a significant effect of diagnostic group. This suggests that much of the variance due to diagnostic group can be attributed to the higher response criterion in children with autism. This is perhaps not surprising on an intuitive level: autism is associated with behavioral rigidity, very literal interpretation of language (Vicker, 2009), and distress when rules are broken (Bolling et al., 2011). These traits may lead to a more conservative criterion to avoid breaking the “rule” that a positive response should only be given if one is absolutely certain the stimulus was present. In other words, for a child with autism, the risk of a false positive may feel higher than the risk of missing a target in the detection task.

Interestingly, the value of *c* across the four subgroups defined by diagnostic status and age was similar for the children with ASD, adults with ASD, and NT adults. This suggests that children with ASD have an unexpectedly mature conservatism in their response bias, relative to their NT peers. Sensitivity, measured by *A*_z_, while significantly lower in autism, was not a significant predictor of threshold, suggesting that the ability to distinguish signal from noise is not a primary factor in light touch detection, and does not contribute to higher thresholds in ASD. In the case of *A*_z_, the group difference was driven by adults with ASD, in contrast to the elevated response criterion that was driven by children and adolescents.

In the whole sample, older age predicted higher thresholds, and this effect was also driven by the adult subsample. The effect of age is consistent with an established literature describing a decrease in tactile sensitivity as adults age (Thornbury and Mistretta, 1981; Kenshalo, 1986; Stevens et al., 2003). This effect survived the addition of *c* to the model, suggesting that it reflects a true decrease in sensitivity rather than a change in criterion.

In the child/adolescent subsample, higher thresholds were associated with parent report of altered reactivity to environmental sensory stimuli broadly as measured by the Sensory Profile (SP), and with global autism symptoms as measured by the Social Responsiveness Scale (SRS). However, these measures share significant variance with the factor of diagnostic group, and thus are likely to be confounded with ASD diagnosis. In support of this interpretation, the correlations were attenuated substantially and were no longer statistically significant when performed within the ASD child/adolescent group alone. While the autism spectrum is continuous with neurotypical development, and thus it is instructive to consider the correlations across the entire sample, the relationships between core autism features, behavioral sensory reactivity, and tactile thresholds do not appear robust. This highlights the complexity of mapping psychophysical measures of sensation and perception (measured with non-ecologically valid, but well-controlled stimuli such as von Frey filaments) onto behavioral measures, for which the response to a light touch stimulus is embedded in a context that includes environmental noise, unpredictability, and multisensory interactions.

Our study had a number of strengths, as well as some important limitations to consider. Among the strengths are a large sample that ranges in age from children through adults, allowing us to discover that differences appear restricted to childhood and adolescence, after which group differences narrow to become negligible. This has important implications for the developmental trajectory of sensory symptoms in autism but is not definitive without longitudinal data. Our paradigm also included null catch trials, which allowed us to calculate sensitivity and response bias and thus to begin to tease out sensory from higher-order explanations. Robust statistical approaches were used to accommodate skewed and/or heteroskedastic distributions of continuous variables. Another strength is the collection of both psychophysical and clinical self/proxy report measures of sensory function, allowing exploration of the links between these two approaches to characterizing sensory ability.

The study is limited by the exclusion of individuals with FSIQ <70 from the sample in order to ensure adequate comprehension of task instructions. A future direction to address this limitation, which is widespread in experimental autism research (Russell et al., 2019), may be to find ways of adapting psychophysical tasks to require less explicit responses in favor of more reflexive or automatic responses. Further, our child/adolescent sample represented a wide age range; future studies should examine more precise developmental periods. Another limitation is the lack of measurement of psychophysical parameters beyond threshold, which could mask important effects either in suprathreshold stimulus processing or in the properties of the psychometric function (Bryant et al., 2019). We did not have a tactile-specific questionnaire measure of behavioral reactivity, which is a common construct of interest for sensory-based therapies. Future studies would benefit from the development and validation of modality-specific questionnaire measures. The clinical implications of this study, however, suggest that, to whatever extent sensory-based or other therapies focus on sensory thresholds as an intervention target or outcome measure, care should be taken to interpret thresholds in light of higher-order cognitive/decisional differences.

Additional future directions include (1) replication with an adaptive staircase method, which would allow for more trials near threshold and thus a more accurate estimation of threshold, (2) replication with the method of constant stimuli, which would complement the staircase by presenting fixed values more randomly and might be a truer estimate of actual threshold, though with the tradeoff of longer testing time, (3) exploration of sensitivity and response criterion in other psychophysical paradigms like the two alternative forced choice method, and (4) determination of other lab-based metrics rather than thresholds that may better map onto clinically significant behavioral reactivity differences to sensory stimuli, and (5) longitudinal, developmental studies of perceptual decision-making.

In conclusion, the current report finds largely intact light touch detection thresholds at the thenar eminence of the palm in adults with autism and corroborates previous findings of elevated thresholds in later adulthood. We also report elevated light touch detection thresholds in children and adolescents with autism. However, further examination of hit and false alarm rates suggests that the primary driver of elevated thresholds in the children with ASD is their unusually conservative response criterion, relative to children with NT. These findings both shed light on basic tactile perception and perceptual decision-making in autism and provide avenues for future experiments to pursue, with an ultimate goal of better understanding the neurobiological and behavioral aspects of altered sensory experiences and translating this knowledge to better treatment approaches.

## Data Availability Statement

The datasets generated for this study can be found in the National Database for Autism Research (NDAR) repository (https://nda.nih.gov/).

## Ethics Statement

The studies involving human participants were reviewed and approved by the Vanderbilt University Human Research Protection Program. Written informed consent to participate in this study was provided by the participants and/or when applicable, by the legal guardian/next of kin.

## Author Contributions

JQ-Z, CO, and ZW performed background reading and synthesis, data cleaning and management, statistical analysis, and drafted sections of the manuscript. LM and BH collected and organized data, read and commented on the manuscript. AW conducted diagnostic evaluation and other clinical and cognitive assessments for the study, read and commented on the manuscript. NW assisted with formulation of hypotheses, read and commented on the manuscript. CC conceptualized the study, formulated hypotheses, supervised data collection and analyses, oversaw drafting and editing of the manuscript.

## Conflict of Interest

The authors declare that the research was conducted in the absence of any commercial or financial relationships that could be construed as a potential conflict of interest.
