# Supplementary material for: Elevated Thresholds for Light Touch in Children With Autism Reflect More Conservative Perceptual Decision-Making Rather Than a Sensory Deficit
Source: Front Hum Neurosci. 2020 Apr 7;14:122. doi: 10.3389/fnhum.2020.00122 (PMC7154145; doi:10.3389/fnhum.2020.00122)
Supplement: Supplementary file 1 [file Table_1.DOCX]

|  | | | |  | |  | | |  | | |
| --- | --- | --- | --- | --- | --- | --- | --- | --- | --- | --- | --- |
|  | | **ASD - Full Sample** | | | |  | **TD - Full Sample** | | |  |  |
| **Hit/**  **Miss** | 0.53 (0.28–0.90) | | 0.47 (0.10–0.72) | |  | 0.65 (0.40–0.92) | | 0.35 (0.07–0.60) |  |  |  |
| **FA/**  **CR** | 0.03 (0–0.25) | | 0.97 (0.75–1) | |  | 0.02 (0–0.25) | | 0.98 (0.75–1) |  |  |  |
|  |  | |  | |  |  | |  |  |  |  |
|  | **ASD - Adults** | | | |  | **TD - Adults** | | |  |  |  |
| **Hit/**  **Miss** | 0.52 (0.32–0.90) | | 0.48 (0.10–0.68) | |  | 0.65 (0.40–0.90) | | 0.35 (0.10–0.60) |  |  |  |
| **FA/**  **CR** | 0.04 (0–0.25) | | 0.96 (0.75–1) | |  | 0.02 (0–0.05) | | 0.98 (0.75–1) |  |  |  |
|  |  | |  | |  |  | |  |  |  |  |
|  | **ASD - Kids** | | | |  | **TD - Kids** | | |  |  |  |
| **Hit/**  **Miss** | 0.55 (0.28–90) | | 0.45 (0.10–0.72) | |  | 0.64 (0.42–0.92) | | 0.36 (0.07–0.57) |  |  |  |
| **FA/**  **CR** | 0.02 (0–0.25) | | 0.98 (0.75–1) | |  | 0.02 (0–0.25) | | 0.98 (0.75–1) |  |  |  |

**Supplementary Table S1.** Signal detection theory matrices for ASD and TD groups, as well as age-specific subgroups. Values indicate proportion of hits (upper left), misses (upper right), false alarms (FA; lower left), and correct rejections (CR; lower right). Values presented as Mean (Min–Max) for the group.
